# Design of a Miniaturized Meandered Line Antenna for UHF RFID Tags

**DOI:** 10.1371/journal.pone.0161293

**Published:** 2016-08-17

**Authors:** Md. Rokunuzzaman, Mohammad Tariqul Islam, Wayne S. T. Rowe, Salehin Kibria, Mandeep Jit Singh, Norbahiah Misran

**Affiliations:** 1 School of Engineering, RMIT University, Melbourne, Victoria, Australia; 2 Dept. of Electrical, Electronic and Systems Engineering Universiti Kebangsaan Malaysia, UKM Bangi, Malaysia; Northwestern University Feinberg School of Medicine, UNITED STATES

## Abstract

A semi-circle looped vertically omnidirectional radiation (VOR) patterned tag antenna for UHF (*919–923 MHz* for Malaysia) frequency is designed to overcome the impedance mismatch issue in this paper. Two impedance matching feeding strips are used in the antenna structure to tune the input impedance of the antenna. Two dipole shaped meandered lines are used to achieve a VOR pattern. The proposed antenna is designed for *23-j224 Ω* chip impedance. The antenna is suitable for ‘place and tag’ application. A small size of *77*.*68×35*.*5 mm*^*2*^ is achieved for a read range performance of *8*.*3 meters* using Malaysia regulated maximum power transfer *of 2*.*0 W* effective radiated power (ERP).

## Introduction

Radio Frequency identification (RFID) system is an automated identification technology that enables the recognition of an RFID enabled target with additional data such as temperature, date of manufacture etc. A tag antenna plays a key role to complete the operation circle of the RFID system. The read range and the angle of readability highly depend on the tag antenna itself. For application e.g. car park, road toll and door security system, long read range, compact size and omnidirectional tag antenna is highly required to achieve ease of accessibility that is not tag orientation dependent. Presently Ultra-high frequency (UHF) RFID chip consist of complex impedance that is capacitive in nature. Only by perfect conjugate matching of the chip impedance, the tag antenna can realize *100%* power exchange with the chip. Therefore, to achieve conjugate matching impedance with the chip, inductive reactance must be introduced to the tag antenna feeding.

Passive UHF RFID tag antennas with omnidirectional radiation have been presented extensively in the literature to date. A passive UHF tag antenna showed in [1] achieves horizontal omni-directional radiation pattern with a circular patch using polyethylene terephthalate (PET) substrate. Capacitive load and inductively coupled feed loop is used to match the antenna impedance conjugate to the tag chip impedance. An equivalent circuit of the impedance matching feed is depicted. However, if the antenna read range needs to be long enough for a specific application with (horizontal or vertical) omni-directional radiation pattern, complicated capacitive load structure is not appealing and dimension accuracy is hard to achieve during bulk production. Another tag antenna with *6 dBic* gain, a dimension of *189 × 127 mm*^*2*^ and thickness *21*.*6 mm* is proposed in [2]. A shorting plate between the patch and the ground plane is used in the design and modified to tune the input impedance of the antenna. The antenna is multilayered with complicated structures. Apart from bulkiness, this antenna exhibits a narrow bandwidth. A loop tag antenna is shown in [3]. The antenna is designed on Styrofoam with dielectric constant of *~1* covering a frequency starting from *902–928 MHz* (North America). Matching stub technique is used to match the input impedance of the antenna. Two supplementary feeding strips are used to excite circularly polarized radiation. Nonetheless, the antenna has a surface dimension of *90 × 90 mm*^*2*^ and near air dielectric constant substrate is used which may cause alteration in antenna performance, given different applications. Also, the simulated reflection coefficient resonance is away from the goal frequency. Complicated impedance matching structure is shown in [4, 5]. In [4] the exhibited antenna has a dimension that extends equally towards the four corners of the square substrate. The arrow shaped hands are symmetrical to the opposite side. Overall the shape of the antenna depicts a quad-pole type configuration. Nonetheless, the antennas are not highly circularly polarized as emphasized by the author. In [5], a meandered structure is attached with the *80*.*9 × 57*.*2 mm*^*2*^ tag antenna. To match with the chip conjugate impedance an inductive winding path is used. The radiation pattern of the antenna follows basic bidirectional antenna radiation shape at both E and H-plane. Nevertheless, *50 Ω* SMA is connected at the place of tag chip to measure the antenna impedance, compromising current leakage at the port of the antenna and the read range is not confirmed. A multi-layered tag antenna with a parasitic radiator and a ground plane connected using via whole is shown in [6]. The antenna shows stable performance at the near field of metallic object. Proximity coupled feeding technique is used to feed the parasitic radiators. Apart from being complex structure, the reflection coefficient response of the antenna is above *-3 dB* within the operating range of UHF RFID. At one of the two resonances within the UHF RFID band, the measured reflection coefficient tends to above *-9 dB*. Quasi-isotropic radiation pattern is depicted in [7] for active tag antenna in the North American band. T-impedance matching method is used to match the impedance with the aid of a bent dipole. The radiation pattern of the antenna seems to agree with the quasi-isotropic pattern at *xz*-plane. However, the UHF tag antenna has a narrow bandwidth performance that involves a ground plane which increases the design complexity. A meander-line tag antenna is shown in [8]. The antenna is loaded with traditional coplanar waveguide inductor and capacitor elements. A detailed analysis is done in terms of different substrates and thickness. Nonetheless, the radiation pattern of the antenna was not taken into account and the design is complicated to be reproduced and it was not achieved accurately in the prototype depicted in the paper due to congested and complex design.

In this paper, a meandered tag antenna is proposed with vertically omnidirectional radiation (VOR) pattern. The antenna uses a unique semi-circle matching structure that not only forms a conjugate match to the impedance of the tag chip, but also is an integral part of the miniaturized radiating elements. The tag antenna structure is simple and the chip feeding line widths are matched to the chip dimensions. The proposed tag antenna can be fabricated using thin FR4 and Polyethylene terephthalate (PET) substrate, making the design cost effective, application oriented, and compatible with modern low-cost tag production techniques. The antenna design process is shown explicitly along with the conducted simulation and experimental measurement for comparison.

## Antenna Design

**Fig 1:**
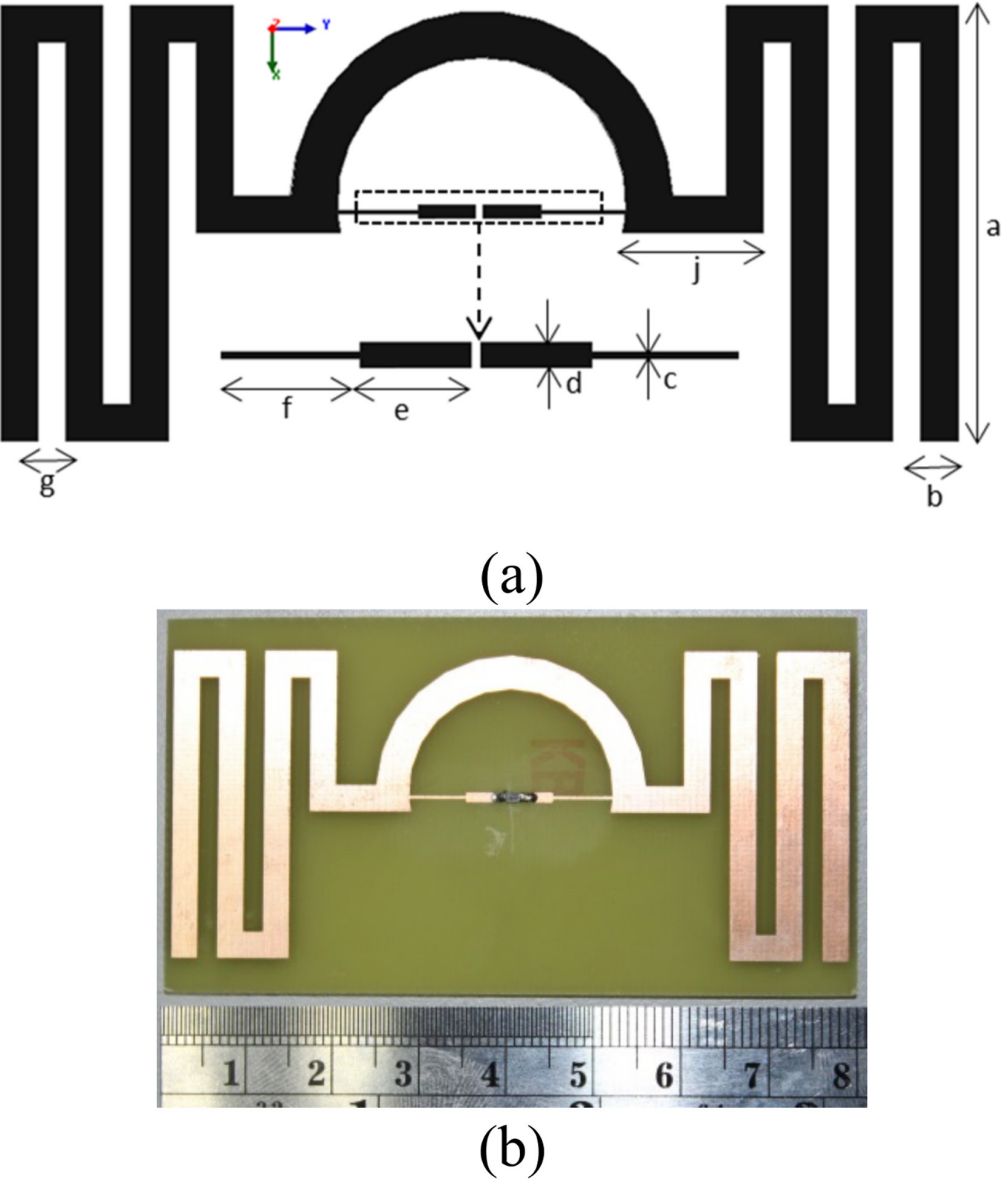
(a) Miniaturized RFID tag antenna geometry, *a = 35*.*5*, *b = 3*, *c = 0*.*3*, *d = 1*.*12*, *e = 4*.*83*, *f = 6*.*52*, *g = 2*.*3*, *j = 11*.*4 (unit*: *mm)*. (b) Fabricated tag antenna.

The geometry of the antenna is shown in Fig 1(A). The proposed tag antenna encompasses a matching semi-circle loop, two chip connecting feed-line strips, and two triple-flipped meandered lines of symmetrical identity at both sides of the antenna. The antenna conductor is constructed using a *35 μm* thick copper over FR4 substrate with a dielectric constant of *4*.*55*, thickness *1*.*6 mm* and loss tangent of *0*.*02*.

The matching semi-circle strip line with a radius of *15*.*5 mm (R)* and *thickness 3*.*8 mm (t)* along with feed-line strip are loaded across the chip. The feed-line strip length can be tuned to match with different impedance of the chip. To match with the selected chips conjugate impedance, the length and width of the feeding strips are optimized to the value of *4*.*83 mm* and *1*.*12 mm* respectively. A bottle neck structure is added at the back end of the feeding strip. A width irregularity is introduced and the width is decreased to *0*.*3 mm* to increase the current density within the structure. The narrow microstrip line travels *7 mm* at both sides to conjugate match the impedance of the chip. The length of the narrow microstrip line can be tuned to match other impedances and hence change the semi-circle radius to connect with the meandered lines. The antenna effective length becomes *352*.*1 mm* after calculating the meandered lines outer boundary, which correlates roughly with the free space wavelength, *λ* at *920 MHz* frequency. The gap between the meandered lines, the line width and length are *2*.*3 mm*, *3 mm and 35*.*5 mm* respectively. The meandered lines establish mutual impedance due to the gap between adjacent lines and the optimized gap is *2*.*3 mm* between the line edges.

**Fig 2:**
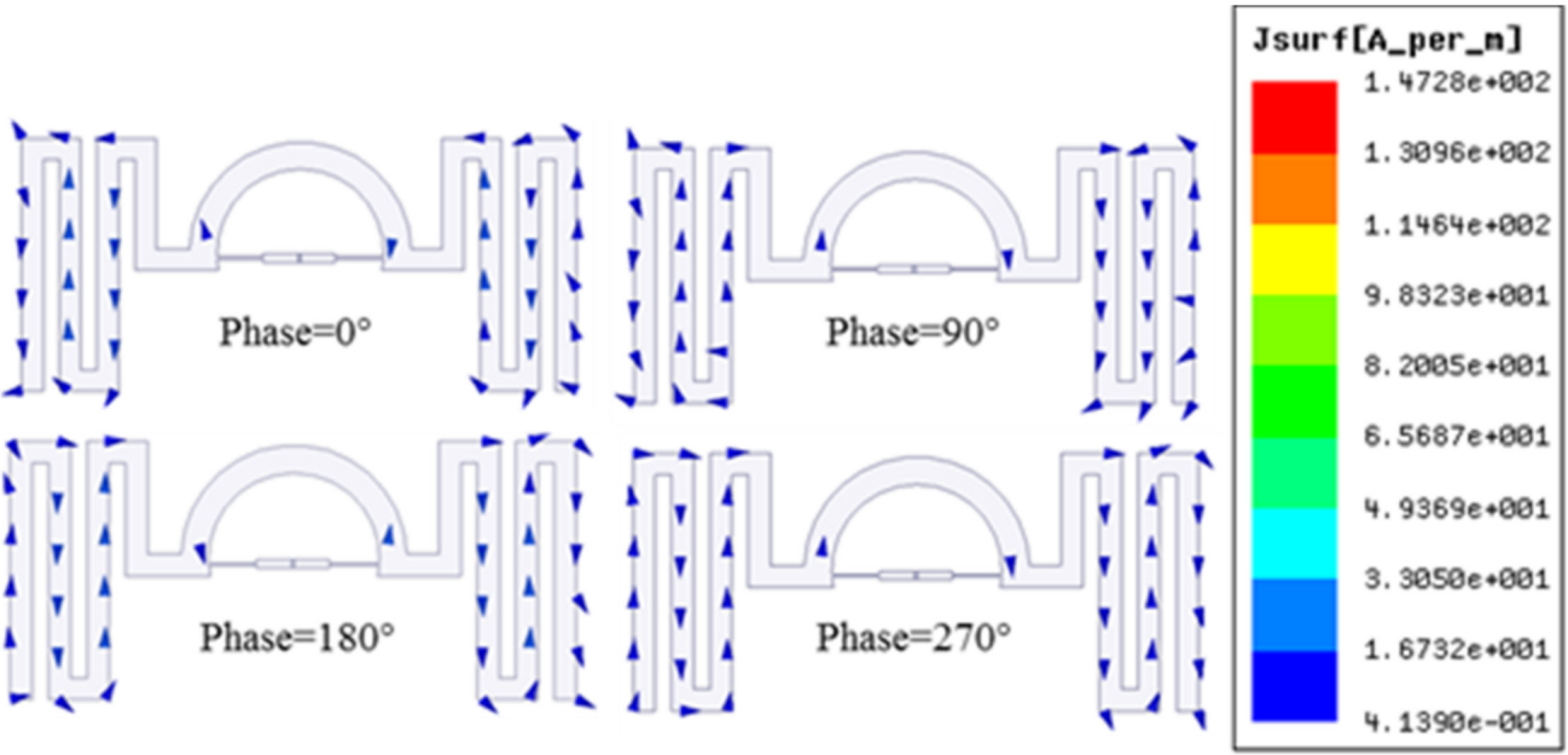
Vector current distribution at different phase at *920 MHz*.

**Fig 3:**
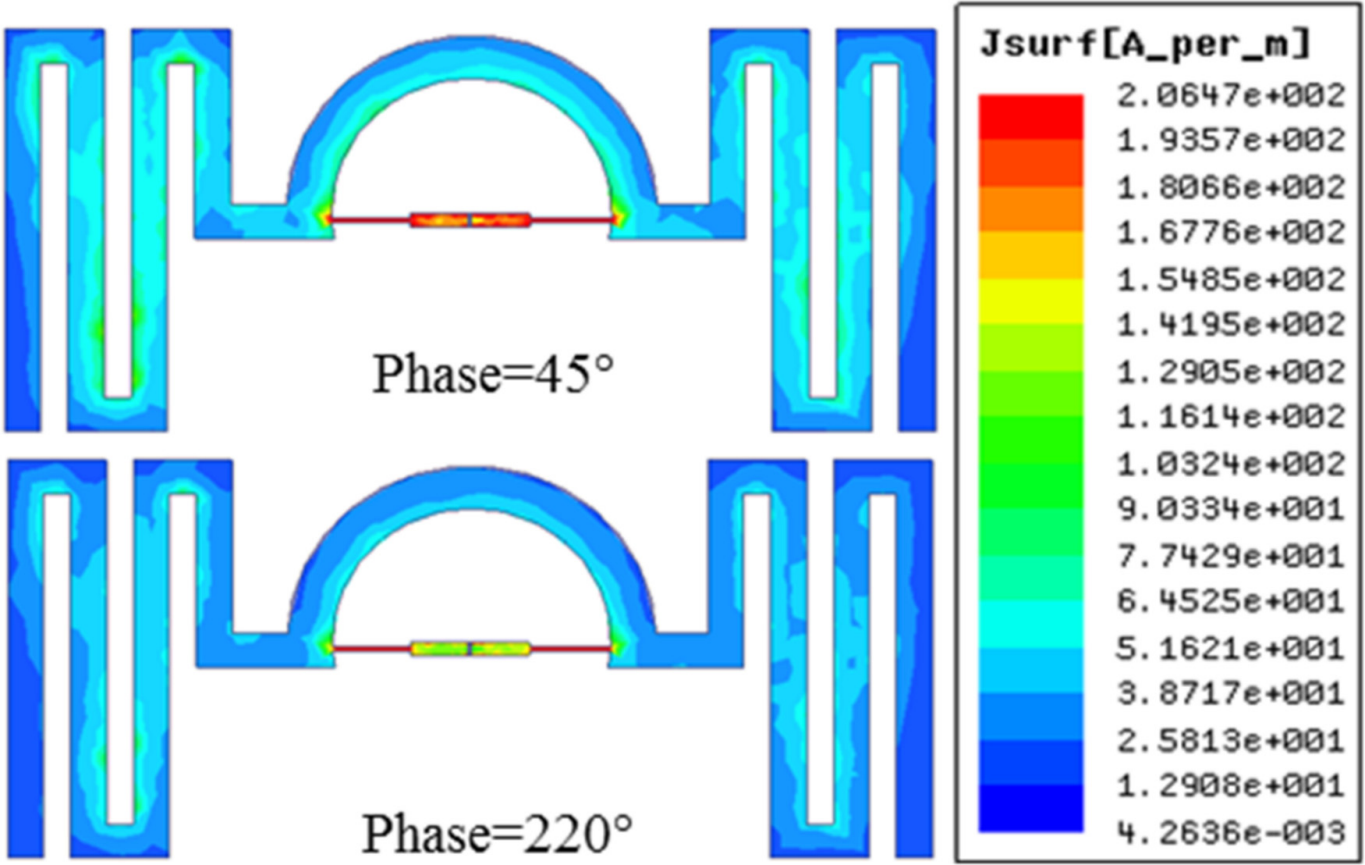
Surface current distribution at *45°* and *220°* phase at *920 MHz*.

The design of the proposed tag antenna is designated to operate in the *919–923 MHz* (Malaysia region) range of UHF RFID band. NXP SL3S1213 UCODE G2iL chip with an impedance of *23-j224 Ω* at *915 MHz* is selected for the tag antenna design. The minimum power to activate the tag chip is *-18 dBm* [9]. Consequently, the input impedance of the antenna must be conjugate matched to this impedance which is *23+j224 Ω* to ensure maximum power transfer between the chip and the antenna. To match the conjugate impedance of the chip, the antenna impedance can be tuned using the thickness (t) of the semi-circle and the gap placement between the feed-line strips. The most effective impedance change is tunable using the width of the chip feeding strip line. By accurately selecting the width and length of the feeding strip and the semi-circle loop, acceptable impedance matching can be realized. The dipoles are meandered at both sides to realize VOR within the operating band. A horizontally omnidirectional radiation (HOR) pattern can also be achieved by adding two more meandered lines perpendicular to the existing meandered lines. Also the semi-circle can be extended to be a full circle to connect the meandered lines perpendicular to each other. Fig 2 shows the current distribution of the antenna at *0°*, *90°*, *180°* and *270°* phase difference respectively. Classic meander line current distribution indicates that vertical segment of the meander line plays the key role to the distribution pattern. The vector current distribution shows that the current is moving from right to left when the phase is at *0°*. When the phase is at *180°*, the current moves from left to right and vacillate perpendicular to the increasing path of the meandered line which results in VOR. Judging the change of vector current distribution for different phase, the current flows counter clockwise. The current induced at the feed points of matching circuit, primarily contributes to the current distribution and vice versa. Fig 3 shows the surface current distribution of the antenna at *45°* and *220°* phase. These two phases are chosen where the current tends to move at the opposite direction (referring to Fig 2). At these two phases the current at the surface of the antenna tends to be most dense with a peak value of *2*.*06e*^*2*^ *A/m*. As the impedance matching circuit is well matched with the conjugate impedance of the chip, the current density tends to be higher at the feed-lines compared to the meandered lines. Nonetheless, the symmetric meander line structures tend to show current density of more than *1e*^*2*^ *A/m* at an average.

**Fig 4:**
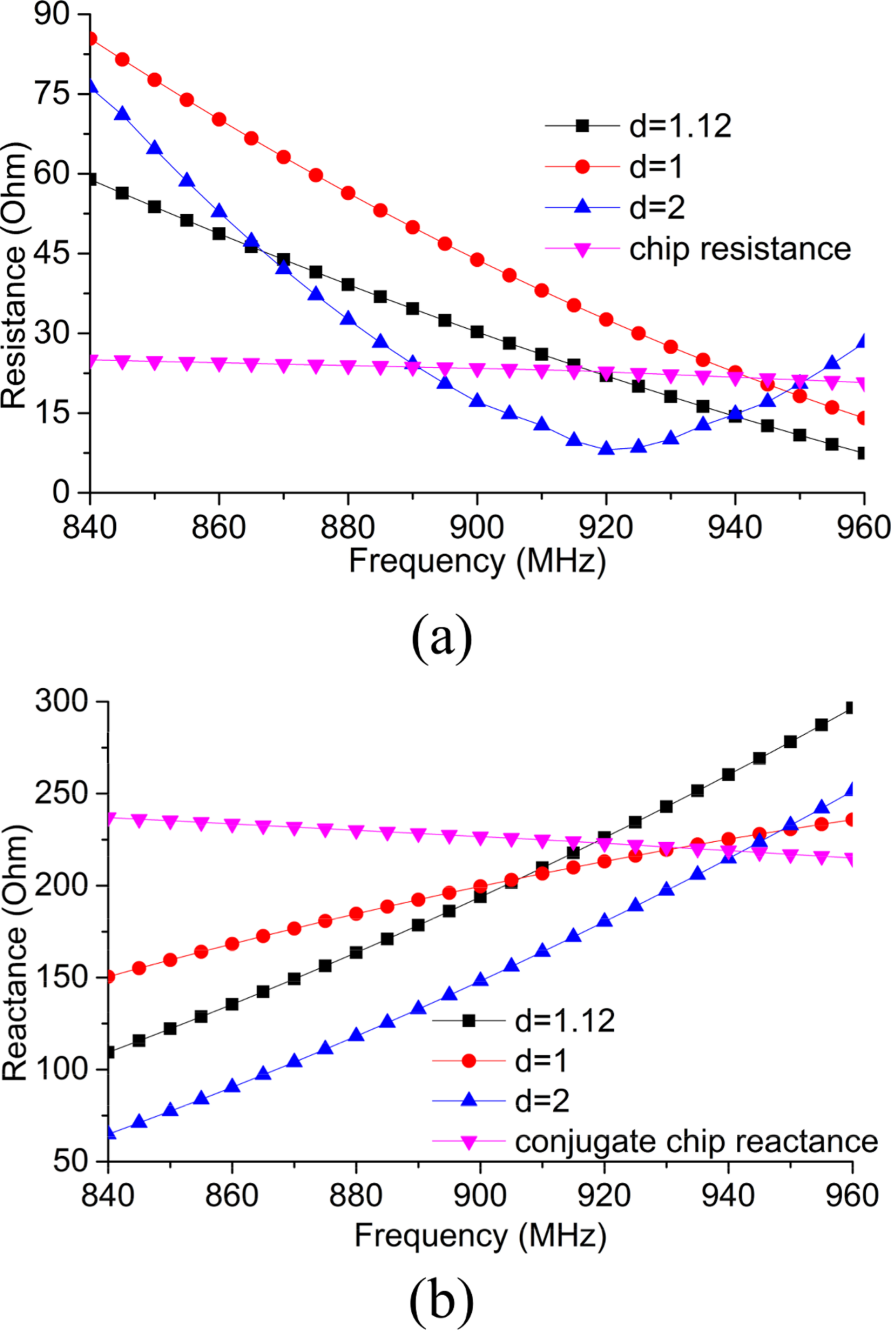
(a) Realized input resistance and (b) realized input reactance at *d = 1*.*12 mm*, *1 mm* and *2 mm* and comparison with the chip conjugate impedance.

To investigate the upshot of the feeding strip lines on impedance characteristics, a parametric analysis is conducted using commercially available HFSS software. The thickness and radius of the semi-circle loop are retained constant. Fig 4(*A*) exhibits the resistance change due to change in the thickness (*d*). As the values of ‘*d*’ changes from *1* to *2 mm*, the antenna resistance varies within the UHF RFID operating frequency (*860–960 MHz*). The intersection between antenna resistance and chip resistance can be observed to have met twice when *d = 2 mm*. However, the desired frequency (919–923 MHz for Malaysia) can be found using the value *d = 1*.*12 mm*. Fig 4(B) manifests the reactance value of the antenna. By comparing the antenna reactance with the conjugate chip reactance, the operating frequency of the antenna can be found to be roughly at the desired frequency when *d = 1*.*12 mm*. To keep the antenna design characteristics symmetrical and improve the antenna measurement process simplicity, no change in the chip position is realized. Nonetheless, by changing the chip placement within the feed-line region, the operating frequency can be tuned for UHF RFID frequency that requires a fixture to measure the impedance performance as depicted in [10]. The measured result of the proposed tag antenna is illustrated in this paper.

## Results and Discussion

**Fig 5:**
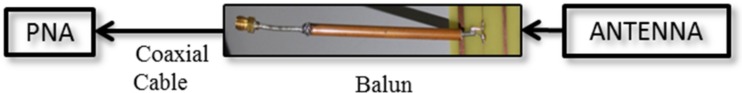
Impedance measurement setup.

The proposed tag antenna prototype is shown in Fig 1(B). For experimental verification, the antenna is measured in an Agilent N5227A power network analyzer (PNA) using balun of *λ/4* length. The setup of the measurement process is shown in Fig 5. The proposed symmetrical antennas feeding points are connected with the balun using its two poles and soldered with fine connection. The other end of the balun is connected with a previously calibrated coaxial cable which is connected to the PNA for the impedance measurement.

**Fig 6:**
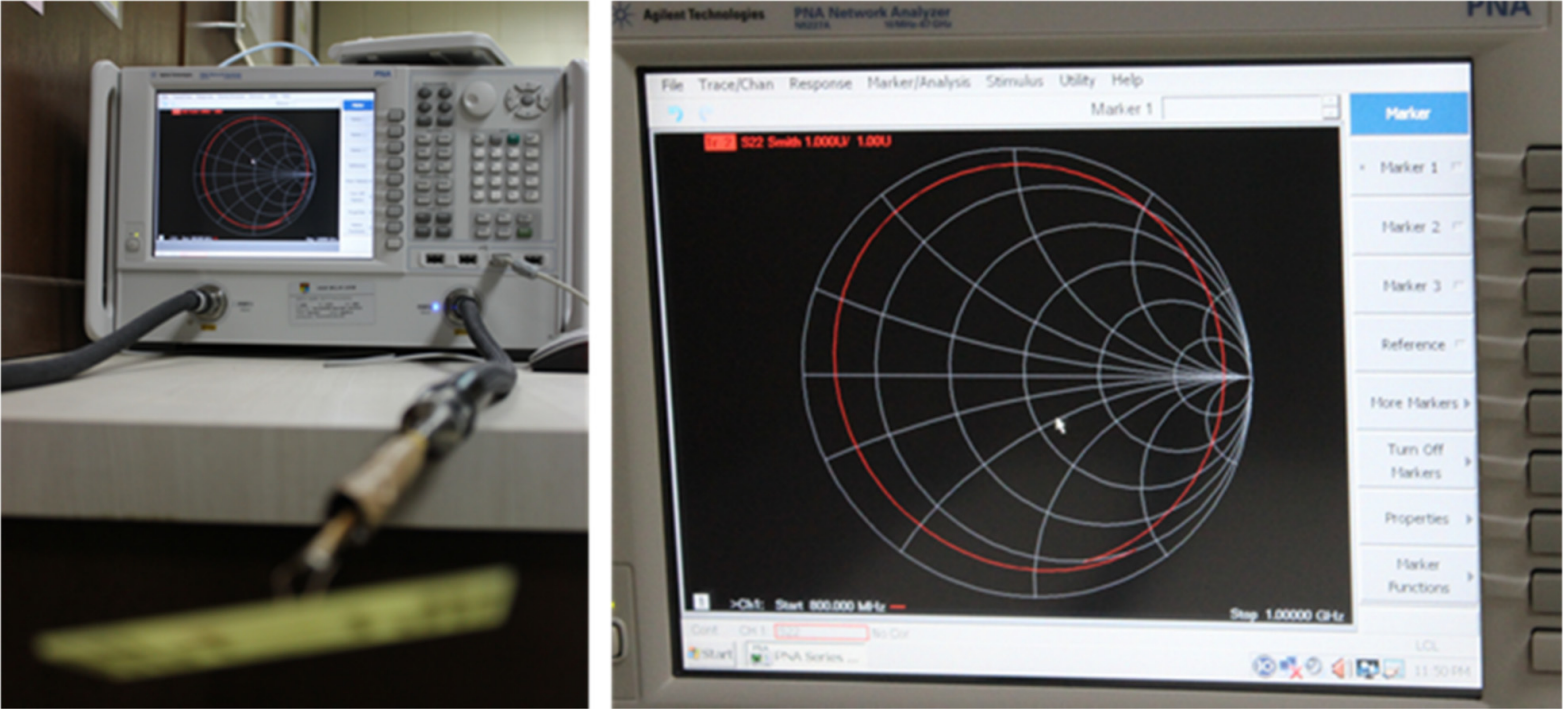
Measured impedance of the antenna using N5227A PNA.

Antenna impedance response depicted in Fig 6 taken at the frequency between *800 MHz* and *1000 MHz*. The smith chart response exhibits that the antenna impedance is within the near field of the conjugate impedance of the tag chip at the desired frequency.

**Fig 7:**
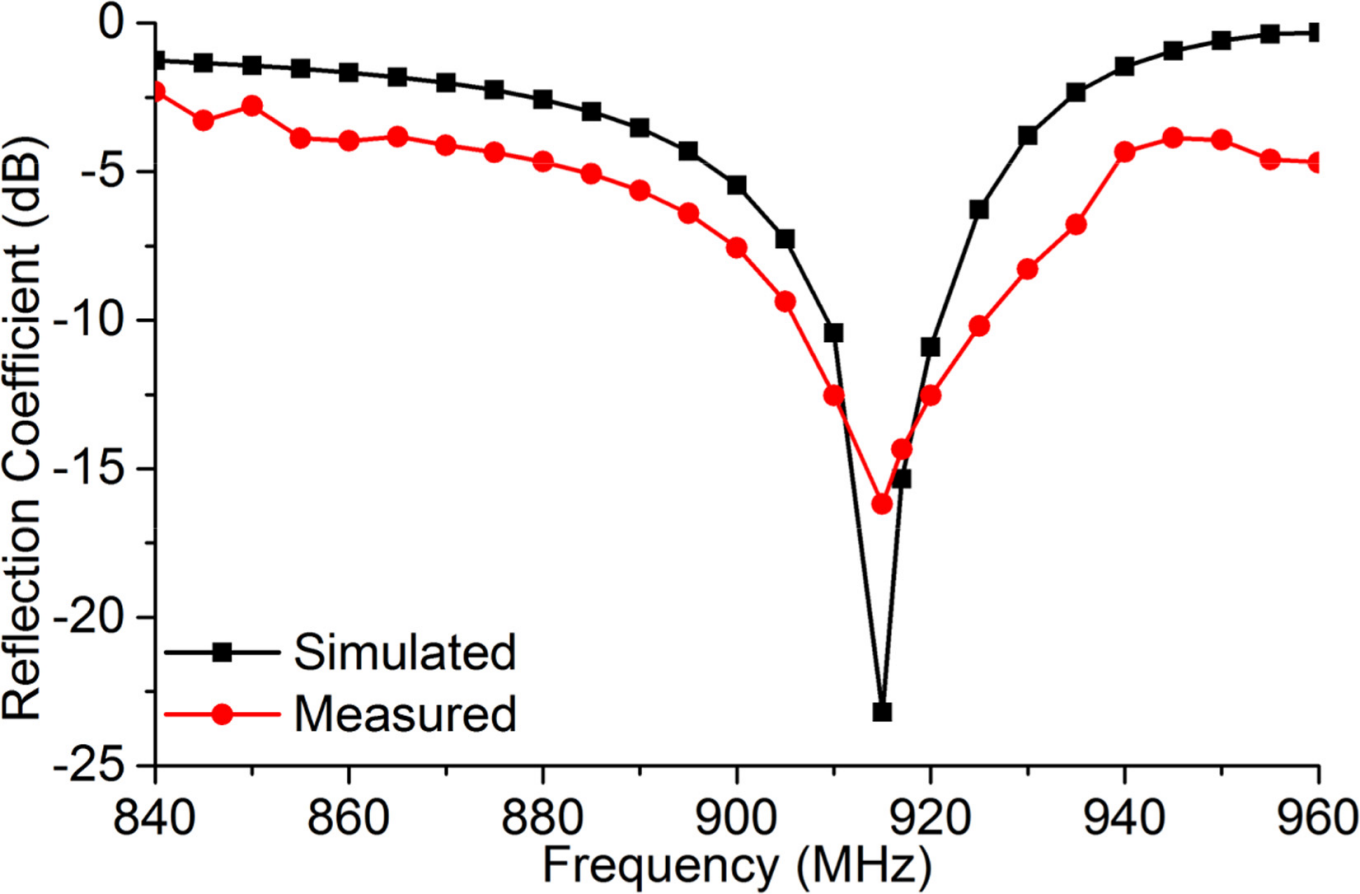
Measured and simulated reflection coefficient of the proposed tag antenna (matched with *23+j224 Ω*).

The measured and simulated reflection coefficient comparison is shown in Fig 7. Although the typical reference impedance *Z*_*0*_ for the Reflection coefficient (*S*_*11*_) is *50 Ω*, in this case, the reference impedance is the tag chip impedance (as the antenna impedance needs to be conjugate matched with the tag chip impedance). The reflection coefficient is calculated from the Resistance and reactance response using the equation given below [4]:

$$S_{11}=\frac{Z_{0}-Z_{a}^{*}}{Z_{0}+Z_{a}}$$ (1)

Where, *Z*_*a*_ is the antenna impedance and Z^*^_a_ is the conjugate value of *Z*_*a*_. Same equation is used to extract the simulated reflection coefficient from Fig 4.

The average of three distinctive measurements is taken for the measured data. The measured data agrees with the simulated data. However, the resonance frequency of the measurement is shifted by approximately *3 MHz* towards the higher frequency compared to the simulated result. It can be caused due to failure in achieving the exact dimension in chemical etching process or balun soldering error. One of the significant reasons behind the mismatching between the simulation and measured result within global UHF RFID frequency range is that, the antenna is simulated for the specific conjugate impedance of the chip at *920 MHz*. The chip impedance changes with the change in the operating frequency. Nonetheless, during simulation process of the proposed antenna, impedances at different frequencies are assumed to be constant. Hence trivial mismatch between simulation and measured result can be observed. In a *-10 dB* reference scale, it can be observed that the prototype antenna achieves a bandwidth of *21 MHz* starting from *907 MHz* till *928 MHz* with a reflection coefficient of *-12*.*5 dB* at *920 MHz*.

**Fig 8:**
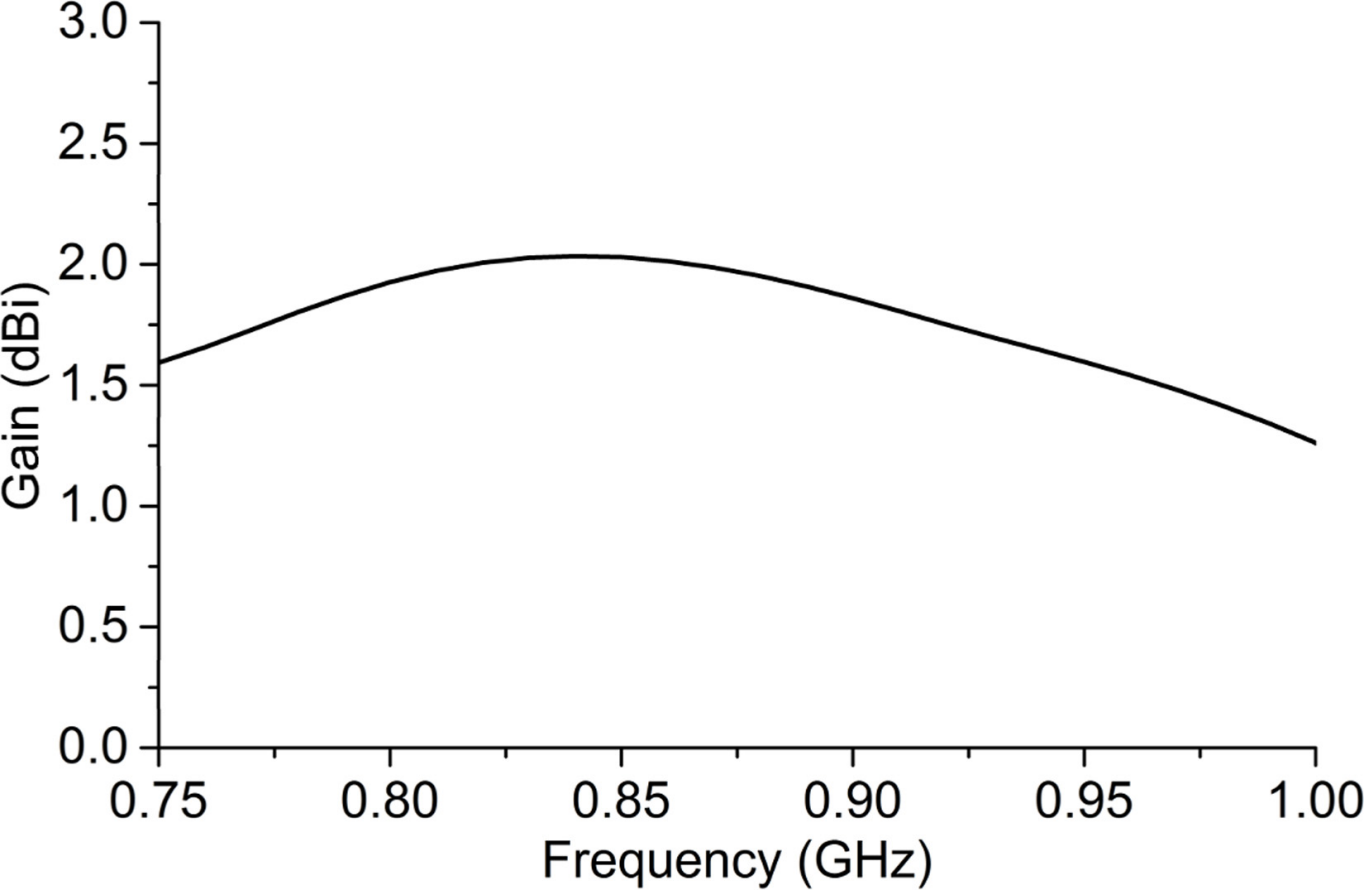
Peak gain of the antenna.

The theoretical peak gain is shown in Fig 8. A stable gain performance is exhibited for the UHF RFID frequency band. At *920 MHz*, a peak gain of *1*.*75 dBi* is achieved for the proposed tag antenna. The effective aperture, *A*_*e*_ of the antenna is calculated to be 0.1194λ^2^ using the equation given below:

$$A_{e}=\frac{\lambda }{4\pi }G$$ (2)

Where, ‘*G*’ is the peak gain of the antenna.

**Fig 9:**
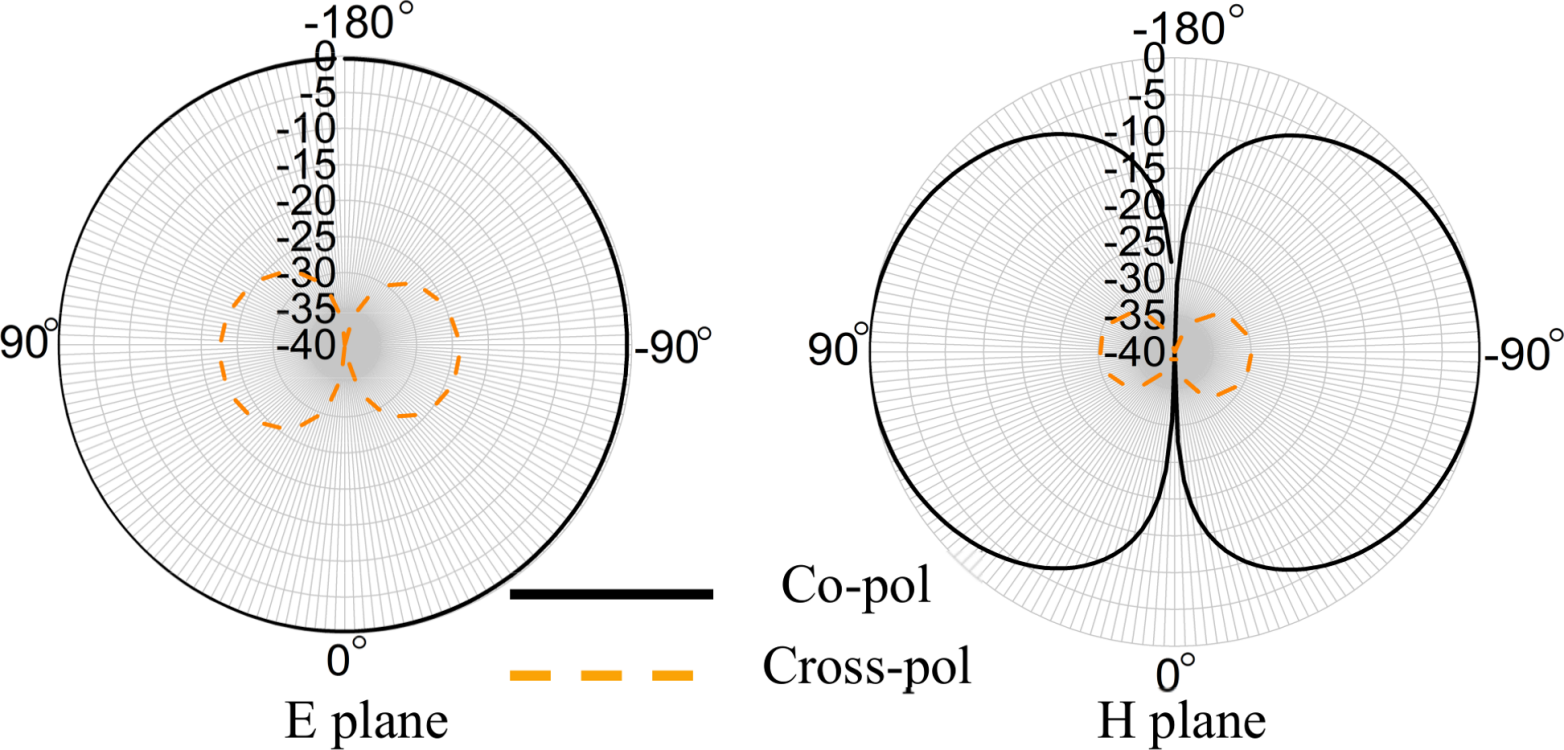
Realized radiation pattern at 920 MHz.

The proposed tag antenna has an impedance of *23+j224 Ω* which is mismatched with the conventional commercially available PNA impedance (*50 Ω*). A method is presented in [11] to measure the radiation pattern of the tag antenna. However, the method involves a Tagformance measurement system. Another method is proposed in [12] that regulate the power by using an amplifier. To keep the simplicity of the design process the realized near field radiation pattern at *920 MHz* is exhibited in Fig 9 at both predominant *E* and *H*-planes. The co-polarized field at *E* plane presents with an omnidirectional radiation pattern. Two null radiating points can be observed in the direction perpendicular to the substrate that is identical to the monopole antennas. The cross-polarization is minimized substantially. Radiation efficiency, ‘*e*’ of the tag antenna can be calculated using the equation as in [10]:

$$e=\frac{S_{IC}}{T_{c}P_{th}\tau D_{m}}$$ (3)

Where, *S*_*ic*_ = chip sensitivity, *T*_*c*_ = transmission coefficient, *P*_*th*_ = threshold power, *τ* = power transfer coefficient and *D*_*m*_ = maximum directivity of the antenna.

**Fig 10:**
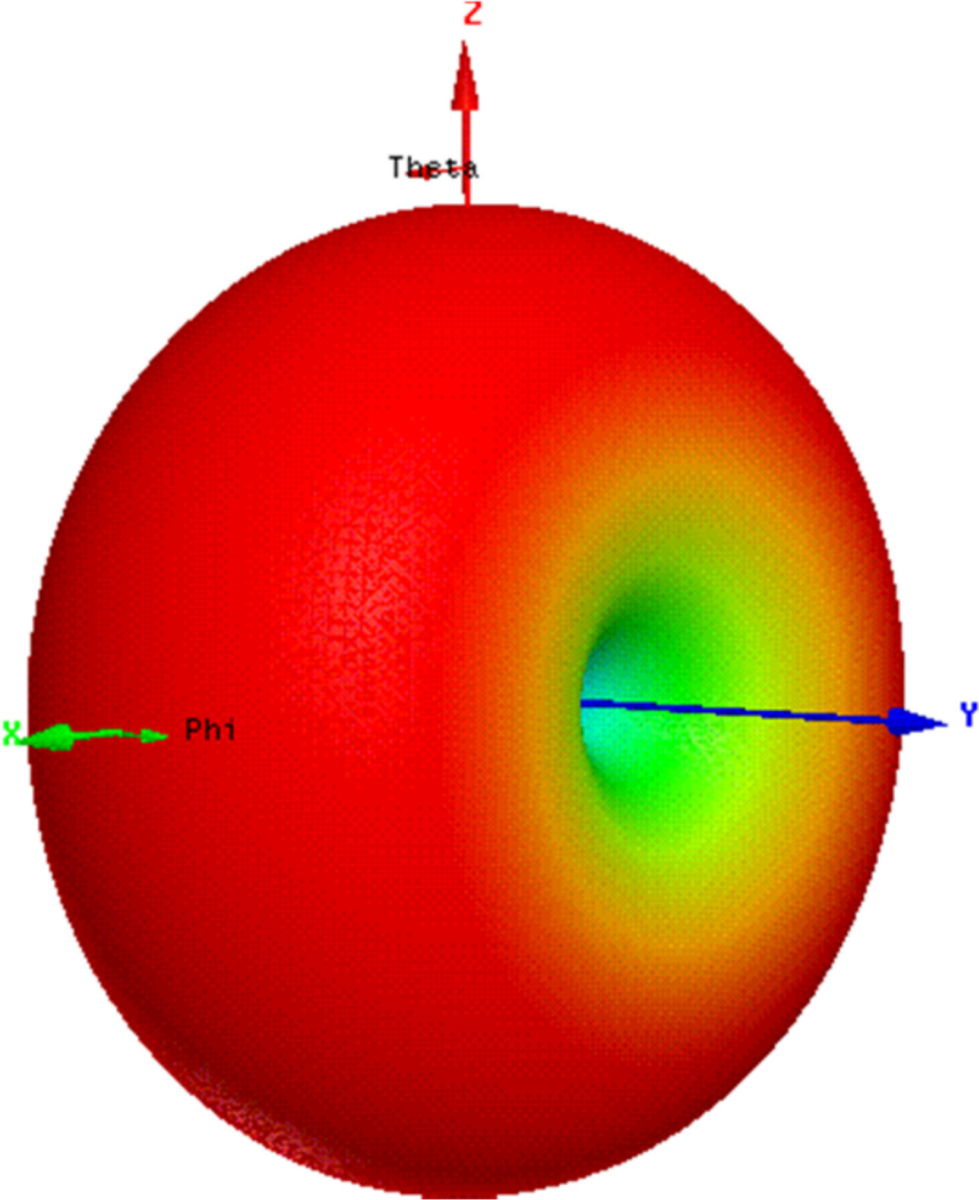
3D radiation pattern of the antenna at 920 MHz.

Fig 10 depicts the combination of the figures shown in Fig 9 in 3D format to understand the real field scenario of antenna radiation pattern.

## Read Range Measurement

**Fig 11:**
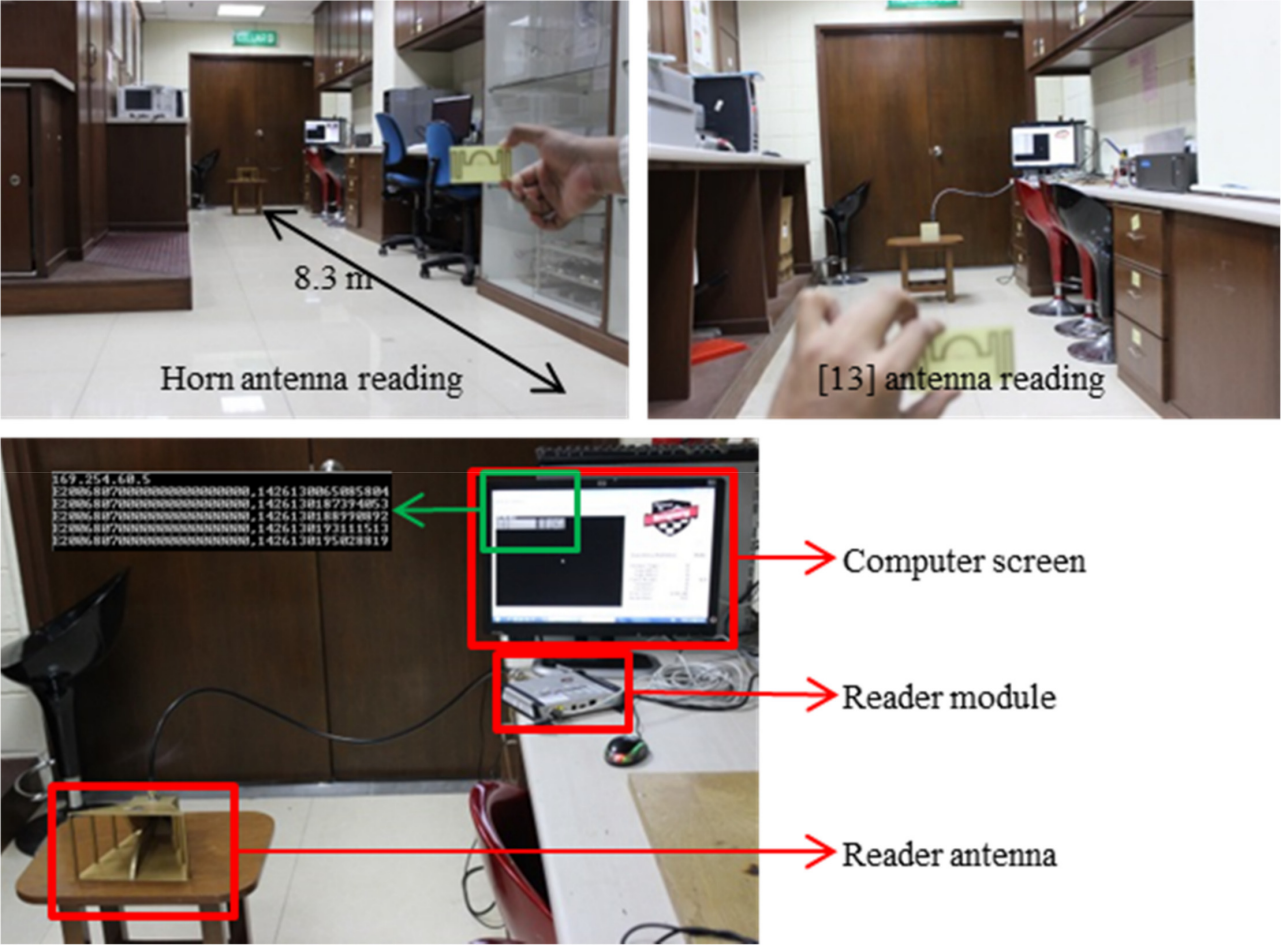
Read range measurement setup in practical environment.

Read range of the tag antenna depends on the application that is mounted on, and long read range is preferred. The measured read range pattern presents with a fundamental criterion of the real scenario antenna radiation pattern. The prototype shown in Fig 1 is attached with the NXP IC chip comprising a sensitivity of *-18 dBm*. To investigate the read range pattern of the antenna the setup is completed using an Impinj speedway revolution R420 UHF RFID reader module, a horizontally linearly polarized horn antenna of model no. *SAS-571* with a gain of *7*.*6 dBi* (after *10 meters* calibration) at *900 MHz* and lastly, the proposed tag antenna. Fig 11 exhibits the measurement setup in practical environment. The horn antenna reading setup is shown at the top left indicating a tag antenna measuring distance of about *8*.*3 meters*. At the top right of Fig 11 the reader antenna proposed in [13] is used to measure the tag antenna read range. At the bottom of Fig 11 a close view of the measurement setup indicating the computer, reader module and reader antenna placement is depicted. The computer screen exhibits that the reader is reading the tag antenna frequently. The antenna can receive both circularly polarized (CP) and linearly polarized (LP) signal. Although CP has less read range compared to LP for the same amount of gain and power, if CP reader antenna is used to read the proposed antenna, the tag antenna can be mounted at any angle and will be able to communicate with the CP reader antenna. To communicate with the linearly polarized reader antenna, the tag antenna needs to be mounted according to the line of polarization of the reader antenna or vice versa. As for the transmission of the tag antenna, after receiving the strength from the inbound signal, the tag antenna gets strong enough to send an outbound signal which is linearly polarized for the proposed antenna. However, this linearly polarized signal can be read by both CP and LP reader antenna as CP supports LP (CP is the combination of linear polarization at both axis). To support the claim, the tag antenna is read using both CP and LP reader antenna as shown in Fig 11.

By using linearly polarized reader antenna, a transition cost, *-3dB* polarization loss factor will be added (ideal linear polarization). Under the regulations provided by Malaysian Communications and Multimedia Commission, to achieve *2*.*0 W ERP (~3*.*2 W EIRP)* transmission power at the end of the transmitting antenna with a gain of *7*.*6 dBi*, *0*.*57 Watt* power is calculated to be the exact. The calculated maximum range, *R*_*max*_ can be found using Friis transmission equation where the gain of the tag antenna, *G*_*t*_ is modified as the equation given below:

$$G_{t}=G_{p}(1-{\left|\Gamma \right|}^{2})\rho$$ (4)

$$R_{max}=\left(\frac{10^{\frac{G_{t}+P_{t}+G_{R}+P_{R}}{20}}}{41.88\times f}\right)1000$$ (5)

Where, *G*_*p*_ = peak gain of the tag antenna, *Γ* = antenna reflection coefficient, *ρ* = polarization loss between the tag and reader antenna, *G*_*R*_ = reader antenna gain, *P*_*t*_ = chip maximum power sensitivity, *P*_*R*_ = transmission power of the reader antenna, *f* = frequency in MHz.

The calculated read range for a linearly polarized (LP) reader antenna is *11*.*34 meters* considering polarization loss and reflection coefficient. However, in reality, the reader antenna has a wider polarization pattern compared to the ideal linear polarization.

**Fig 12:**
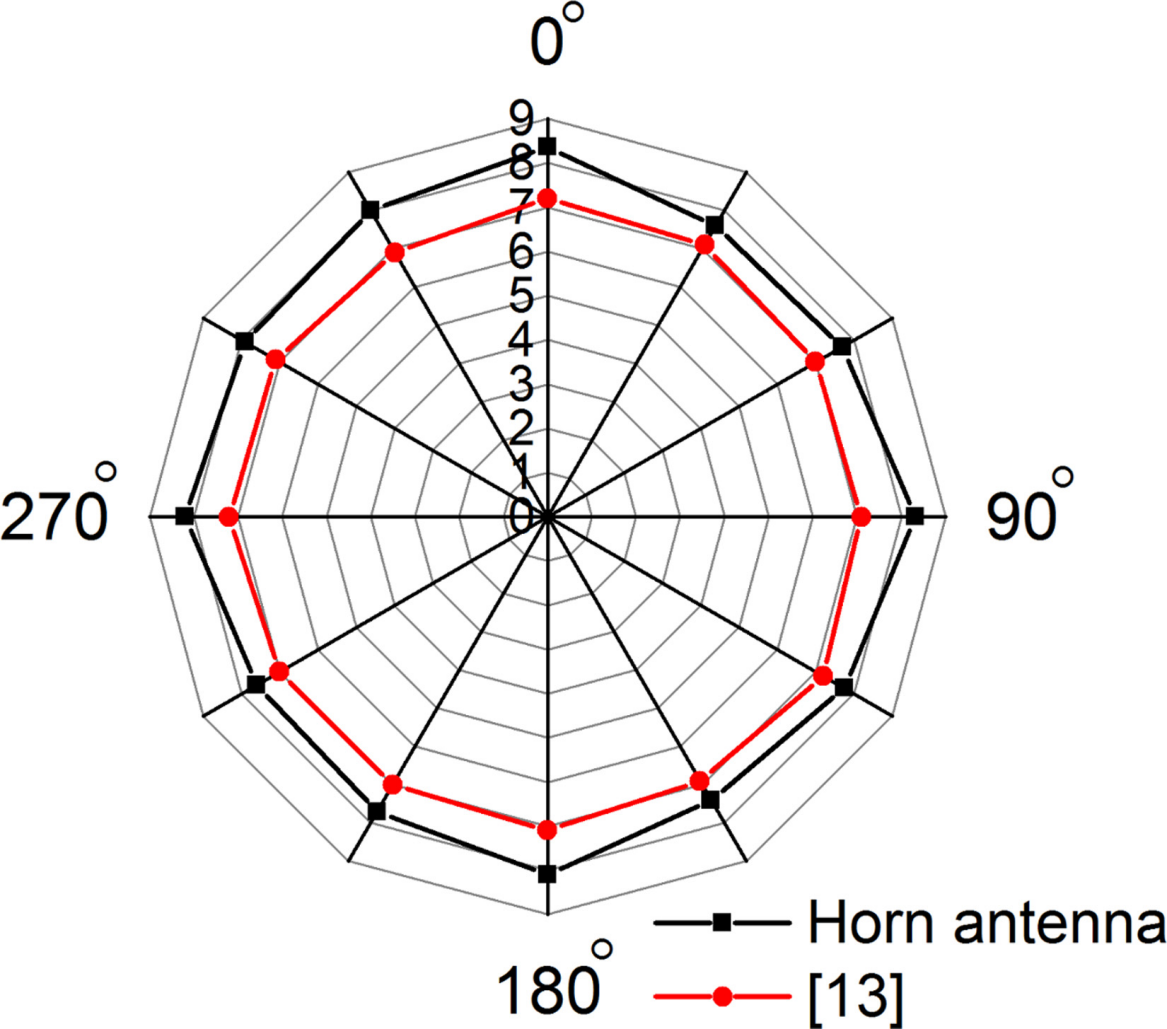
Measured read range with LP reader antenna at zx-plane (E-plane).

The measured read range pattern is shown in Fig 12. The pattern shows the reading at the *zx*-plane. A highest read range of *8*.*3 meters* is found towards the *zx* measurement plane of the tag antenna. Compared to the calculated read range, the measured read range is smaller. The decrement could be due to the polarization loss is more than expected. One impactful disturbance may occur at the connection between the antenna and the chip which in practical, is mounted using industrially available automated circuit designing systems. For the case depicted in this paper, the chip mounting was achieved by using the means available within the facility it is measured in. The sensitivity of the tag chip was taken to be *-18 dB* which might not be the case in practical measurement. No report has been published so far measuring the sensitivity of the chip while receiving power.

Table 1 depicts the comparison between the antennas mentioned in the literature in terms of dimension and substrate of choice. It can be observed that the proposed antenna substrate is chosen to be one of the most popular choices for tag antenna design. Moreover, the proposed tag antenna can be designed using PET substrate. The tag antennas reported here has wide read range including the proposed tag antenna. However, the power fed to the reader antenna varies with the designs mentioned in the literatures. Comparatively, the proposed antenna is read using the lowest EIRP regulated by Malaysian government. With the power increased, the antenna reading range will also increase hence, the proposed tag antenna will be detectable from greater distance. It can be observed from the table that the circular polarized tag antennas can be detected from far distance easily compared to the linearly polarized antennas.

**Table 1:** Tag antenna specifications from the literature.

Antenna	Substrate	Dimension (mm)
[2]	FR4 (Ԑ_r_ = 4.6)	189.6×127.9×21.6
[6]	FR4 (Ԑ_r_ = 4.6)	28×14×3.2
[8]	FR4 (Ԑ_r_ = 3.7)	47.13×14.81×1.6
[12]	Rogers RB 4350B	42×25.7×1.524
**Proposed Antenna**	**FR4 (Ԑ**_**r**_ **= 4.6)**	**77.68×35.5×1.6**

## Conclusion

In this paper, a VOR antenna is studied and implemented for UHF RFID tag application. The tag antenna comprises of a chip with *23-j224 Ω* impedance. A maximum impedance matching of *21 MHz* (*907–928 MHz*) is measured within UHF RFID band using inexpensive FR4 substrate. A maximum peak gain of *1*.*75 dBi* is achieved. The read range of the antenna is measured to be *8*.*3 meters*. *2*.*0 W ERP* power is applied to the reader antennas to achieve these performances. By changing the width of the feed-line structure, the proposed antenna can achieve conjugate impedance matching at different frequencies of UHF band. The VOR antenna gives an efficient and compact solution for Malaysian UHF RFID tag antenna system.
